# Development of a handheld chlorophyll content detector on wheat and maize leaves based on RGB sensor

**DOI:** 10.3389/fpls.2025.1606413

**Published:** 2025-07-11

**Authors:** Weidong Pan, Heng Ma, Rui Wang, Hongrui Wang, Dong Wang, Wenchuan Guo, Xiangkai Guo

**Affiliations:** ^1^ College of Mechanical and Electronic Engineering, Northwest A&F University, Xianyang, Shaanxi, China; ^2^ Xi'an Hanpule Science and Technology Co., Ltd., Xi'an, Shaanxi, China

**Keywords:** chlorophyll, chlorophyll-a, chlorophyll-b, color indices, crop, detector

## Abstract

Chlorophyll-a (CL-a) and chlorophyll-b (CL-b) are major chlorophyll found in green plants. Determining the CL-a, CL-b, and total chlorophyll (TCL) contents is important to guide crop growth. However, the widely used portable chlorophyll detectors, such as SPAD-502, are limited to measuring relative chlorophyll content and can't measure the contents of CL-a, CL-b, and TCL. It was reported that the chlorophyll content was related to the color indices of leaves, which inspired us to develop a portable detector that can non-destructively measure the contents of CL-a, CL-b, and TCL based on a color sensor. Therefore, the world's major crops, i.e., wheat and maize, are used as samples to develop a handheld chlorophyll content detector for leaves in this study. The detector was mainly composed of a microcontroller, RGB sensor, light source, and power management module, etc. The software, developed in Keil μVision5, was composed of a main function and several sub-functions, such as the leaf color collection sub-function, data processing sub-function, key sub-function, and display sub-function. The relationships of CL-a, CL-b, and TCL contents with the color features of wheat and maize leaves were analyzed. The results showed that these chlorophyll contents had high correlations with *B* (blue), *B*′ (blue light intensity), *H* (hue), *S* (saturation), and *V* (value) and can be expressed by five-variable equations. Compared with the chlorophyll contents measured by the traditional spectrophotometry method, the root-mean-square errors of the developed detector were 0.269 mg/g, 0.089 mg/g, and 0.350 mg/g for CL-a, CL-b, and TCL contents, respectively. The small size, light weight, and quick measurement (about 2 s) make the detector will be important for instructing crop breeding, fertilization, and other management.

## 1 Introduction

Chlorophyll is a crucial pigment found in plants, algae, and cyanobacteria. The main function of chlorophyll is to absorb sunlight and convert it into energy through photosynthesis. Chlorophyll-a (CL-a) and chlorophyll-b (CL-b) are the two major chlorophyll found in green plants. CL-a absorbs light in the violet-blue and reddish-orange end of the spectrum and reflects green-blue light, giving the plant a green color. CL-b absorbs light in the blue and red-orange wavelengths. Low chlorophyll content means that the plant has poor photosynthesis. Therefore, detecting the chlorophyll content of plant leaf is routine work in agriculture management, especially in diagnosing disease and fertilizing (Houles et al., 2007).

Spectrophotometry is the standard method for leaf chlorophyll content detection (Chinese Agricultural Industry Standards, 2017, China). Although this method is highly accurate, it has several shortcomings, such as a complex detection process, destroyed samples, and the usage of a hazardous chemical reagent (acetone). To enable quick, non-destructive detection of chlorophyll content in plant leaf, chlorophyll detectors like SPAD-502 (Konica Minolta, Japan) and the MultispeQ multi-function plant meter (PhotosynQ Corporation, USA) were developed. These detectors, however, cannot directly measure chlorophyll content but provide a relative value, such as the SPAD (Soil and Plant Analyzer Development) value. Numerous studies indicate that the relationship of SPAD with the chlorophyll content of different plants or with the chlorophyll content of a single plant at different growth stages is not a constant (Bavec and Bavec, 2001; Hoel, 2003; Le Bail et al., 2005; Schepers et al., 1992). Consequently, it is impossible to accurately predict plant leaves' chlorophyll contents based solely on the SPAD value.

In order to find a method for non-destructive and efficient detection of leaf chlorophyll content, visible/near-infrared spectroscopy (Guo et al., 2014; Xie et al., 2007), fluorescence (Das et al., 2023; Kalaji et al., 2018; Netto et al., 2005), hyperspectral imaging (Zhang et al., 2023a, Zhang et al., 2023b; Zhao et al., 2023), remote sensing (Jay et al., 2017; Pan et al., 2023; Zhang et al., 2019) have been used to predict chlorophyll content of plant leaves. However, these methods generally have the defects of expensive instruments and professional spectra analysis, resulting in these methods not being widely used. Moreover, the measurement results are not true chlorophyll content, such as SPAD, etc., or the predicted chlorophyll content is at the canopy scale using remote sensing technology. To make the chlorophyll content can be detected quickly and non-destructively, Yang et al. (2019) developed a portable chlorophyll detector for a few varieties of leafy vegetables using a photoelectric sensor to sense the transmitted light by two light-emitting diodes (LEDs). However, it just detects TCL content. Different plants have different textures, and the selected wavelengths for the specified plant varieties cannot be used for others, causing poor versatility. Previous studies have demonstrated a significant correlation between chlorophyll content and color characteristics of plant leaves. For example, Li et al. (2012) reported that the combination of green and blue indices exhibited a strong correlation with the chlorophyll content of maize leaves. Zhou et al. (2018) found that the green index of wheat leaves had the highest correlation with SPAD values. To facilitate non-destructive detection of chlorophyll content in plant leaves, Li et al. (2021) proposed a universal method to detect the chlorophyll content of green plant leaves using different smartphones based on obtained RGB (Red, Green, and Blue) images, and they proposed a chlorophyll prediction model for sugarcane leaves based on the indices of blue, green and red. However, a portable auxiliary shoot device with a built-in active light source to reduce the influence of environment was needed when capturing images. These studies inspired us to develop a handheld chlorophyll content detector based on an RGB sensor.

Wheat and maize are significant crops worldwide, but it is unclear if a single model can adequately represent the relationship between their chlorophyll contents and color features. Further, it is also uncertain if it is possible to develop a detector that can determine CL-a, CL-b, and TCL contents based on color features. Therefore, the aims of this study are: (1) to analyze the relationship between the CL-a, CL-b, and TCL contents with the color features of wheat and maize leaves; (2) to develop a handheld chlorophyll content detector using an RGB sensor; and (3) to evaluate the performance of the developed detector.

## 2 Detector's development

### 2.1 Hardware design

**Figure 1:**
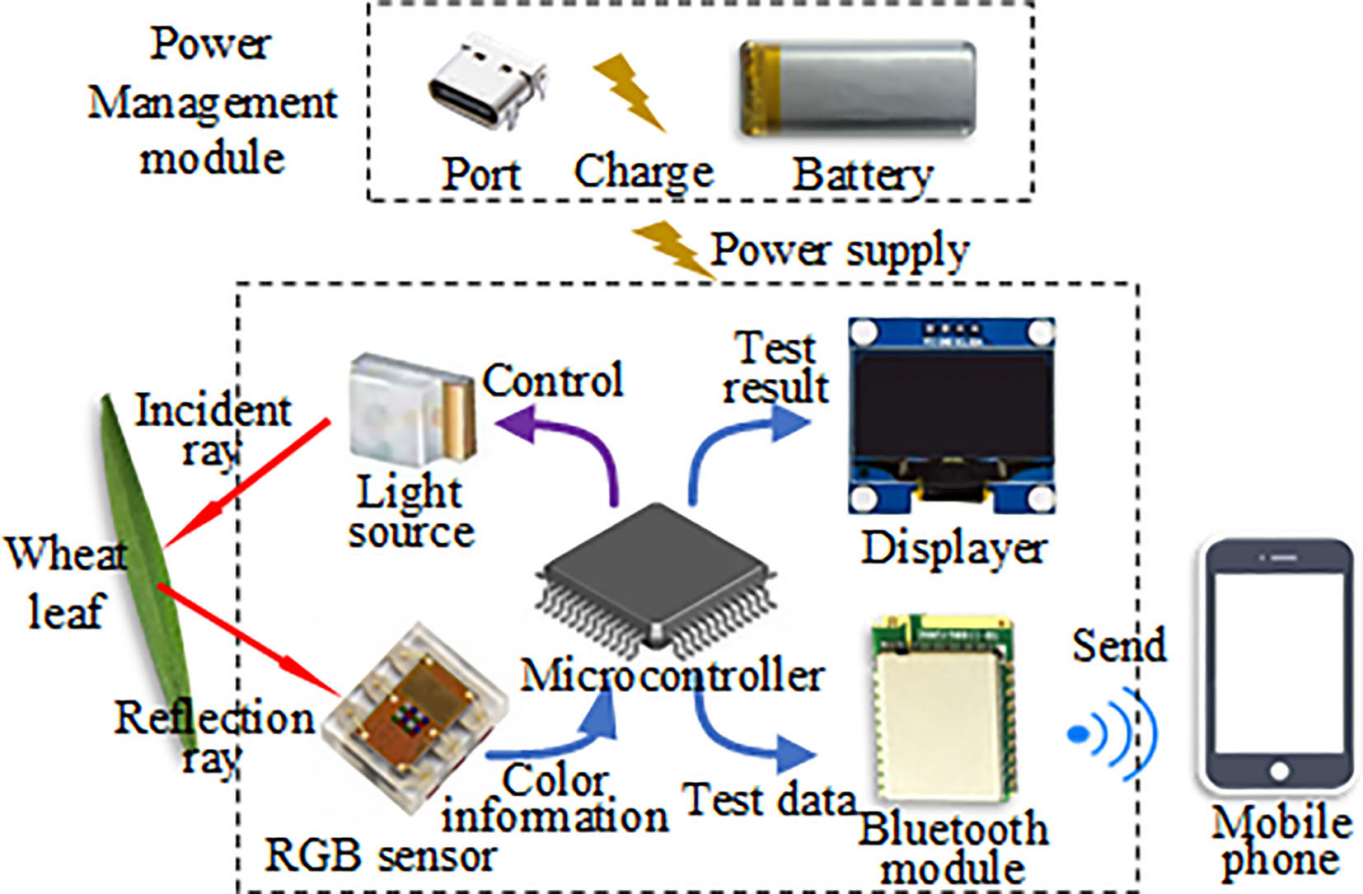
The block diagram of the hardware system for the handheld leaf chlorophyll content detector.

**Figure 1** is the block diagram of the hardware system for the handheld chlorophyll content detector of wheat and maize leaves. The detector comprises a microcontroller, light source, RGB sensor, displayer, Bluetooth module, and power management module. The microcontroller undertakes the task of managing each module. The light source is employed to provide stable light, while the RGB sensor collects the RGB color information of leaves and forwards the data to the microcontroller. The displayer exhibits the detection results. The Bluetooth module enables data transmission to a mobile phone, and the power management module ensures power provision for the entire detector, managing charging functions and offering protection. Lastly, the Type-C interface facilitates serial communication for the microcontroller, and the power charging interface is also used.

#### 2.1.1 Microcontroller

An STM32F103C8T6 chip is used as the microcontroller, which includes an embedded high-speed memory, a high-performance M3 32-bit RISC CPU running at 72 MHz, an enhanced I/O port and peripheral devices. Its operation voltage is 2.0 V-3.6 V, the flash memory capacity is 64 KB, the RAM capacity is 20 KB, and the operating temperature is -40°C-85°C.

**Figure 2:**
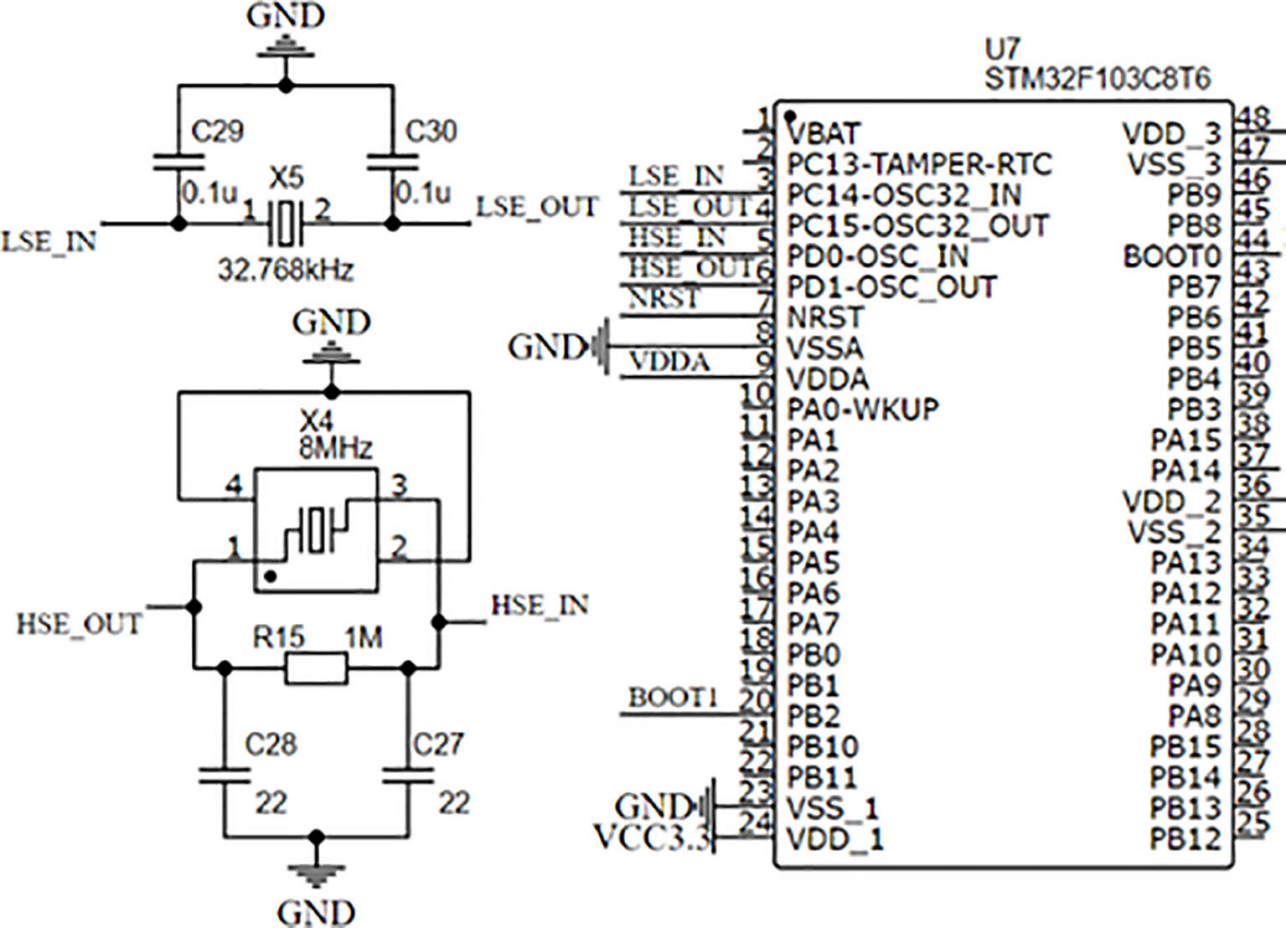
The minimal peripheral circuit of STM32F103C8T6.

**Figure 2** is the minimal peripheral circuit of STM32F103C8T6. The high-speed external crystal vibration is a passive patch crystal vibration of 8 MHz, which provides the main frequency of 72 MHz after the frequency divider and phase-locking ring. The low-speed external crystal vibration is 32.768 kHz, mainly used to provide a stable clock signal for the real-time communication clock of the main control chip.

#### 2.1.2 Light source

In order to avoid the interference of external ambient light in obtaining color information of leaves, the internal light source is used to illuminate the leaves. Considering the light source's luminous color, brightness, and stability, the positive white patch light-emitting diode (LED) of 0603 is applied. It has a color temperature of 6000–7000 K, a luminous intensity of 500 mcd, a maximum forward current of 20 mA, and a maximum power consumption of 70 mA. Its forward current is almost positively proportional to the relative light intensity. Two LEDs are used to ensure uniform illumination. The light source and the sensor are in a horizontal line, symmetrically distributed on both sides of the sensor.

#### 2.1.3 RGB sensor

Considering the performance of the RGB sensor and the cost of the handheld chlorophyll content detector of crop leaf, TCS34725 (ams OSRAM, Vienna, Austria) is used as the RGB sensor. The sensor includes a 3×4 photodiode array that detects the surface color of an object through optical sensing. It outputs the light intensity of red (*R*′), green (*G*′), blue (*B*′), and clear (*C*). The I^2^C protocol is used to communicate between the sensor and the microcontroller.

#### 2.1.4 Displayer and buttons

The displayer is a 0.96-inch organic light-emitting diode (OLED) with a resolution of 128 pixel×64 pixel and a working voltage of 3.3 V. The communication between the OLED and microcontroller is I^2^C. There are three buttons in the detector: "Detection", "Flip", and "Transmission". When the "Detection" button is pressed, the detector starts to detect. When the "Transmission" button is pressed, the measurement results are transmitted to the mobile phone through Bluetooth. The "Flip" button switches display content, that is, CL-a, CL-b, and TCL contents.

#### 2.1.5 Type-C interface module

The Type-C interface is used as the serial communication interface and power charging interface of the detector and personal computer. It has 16 pins symmetrically distributed on both sides to ensure the positive insertion of Type-C terminals. The main pins are DP and DN communication pins and the power supply pin of VBUS. DP and DN pins are connected to the CH340X chip, a USB bus conversion chip, realizing the USB signal turns to the serial port signal.

#### 2.1.6 Bluetooth module

Bluetooth module is used to save the detection data to the mobile phone, and the selected Bluetooth module is DX-BT04-E (Shenzhen Daxia Longque Technology Co., LTD., Shenzhen, China). It integrates the RTL8762 Bluetooth chip and peripheral circuit, supporting SPP V3.0 and BLE V4.2 protocols. The chip has a maximum communication distance of 50 m and communicates with the microcontroller through the asynchronous serial communication mode.

#### 2.1.7 Power management module

**Figure 3:**
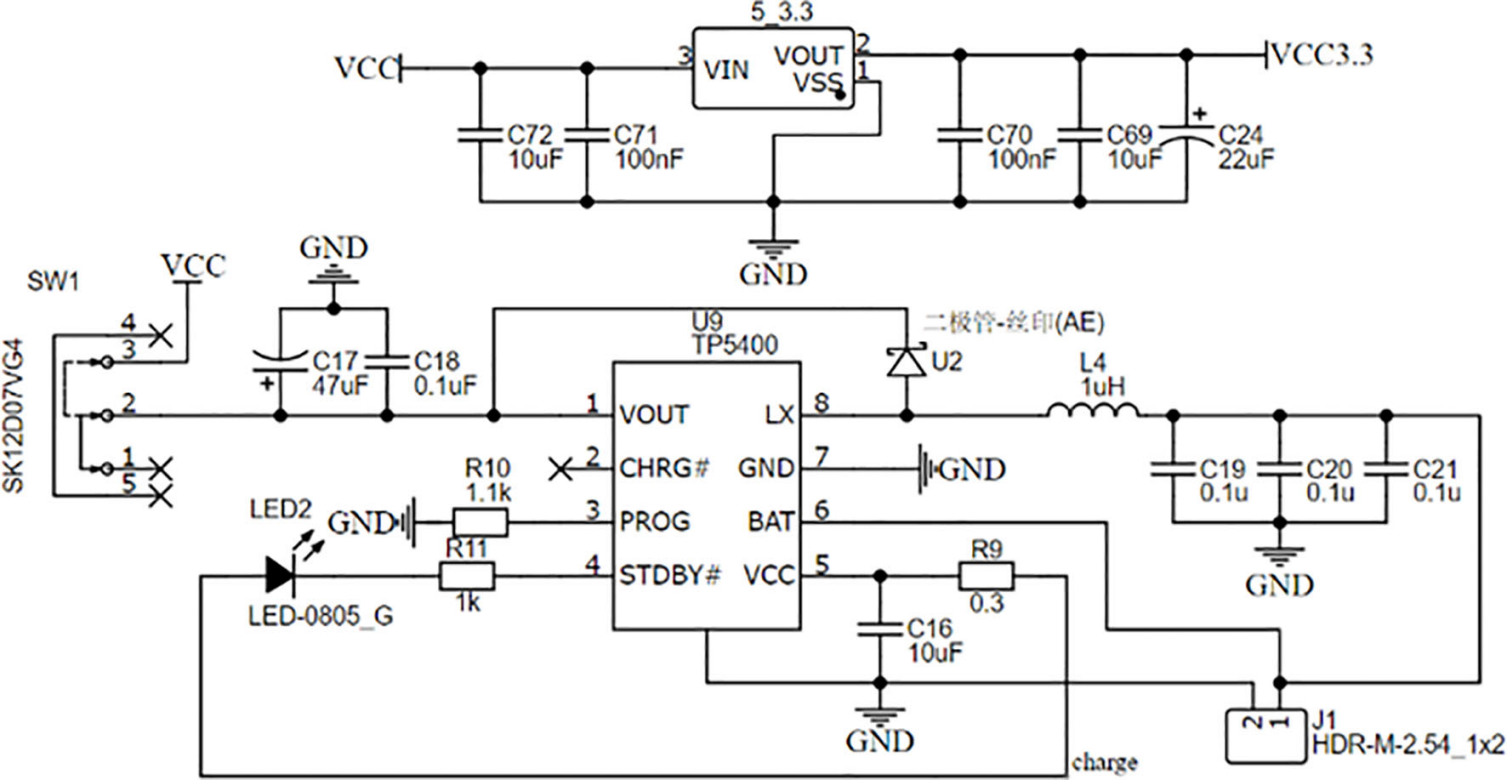
The circuit of power management module.

The power supply of the detector is a 1500 mAh lithium polymer battery with 3.7 V of rated voltage and 1000 mA of rated charging current. The TP5400 chip, which has the functions of charge, discharge, boost conversion, and power indication, is used to manage the power supply. VIN, the charging input of TP5400, is connected to the TYPE-C interface to charge the battery. The boost input BAT is connected to the battery's positive electrode, and the output VOUT is responsible for providing the voltage of 5 V to some components and then reducing the voltage of 5 V to 3.3 V to power other components. The circuit of the power management module is shown in **Figure 3**.

#### 2.1.8 Prototype of the detector

**Figure 4:**
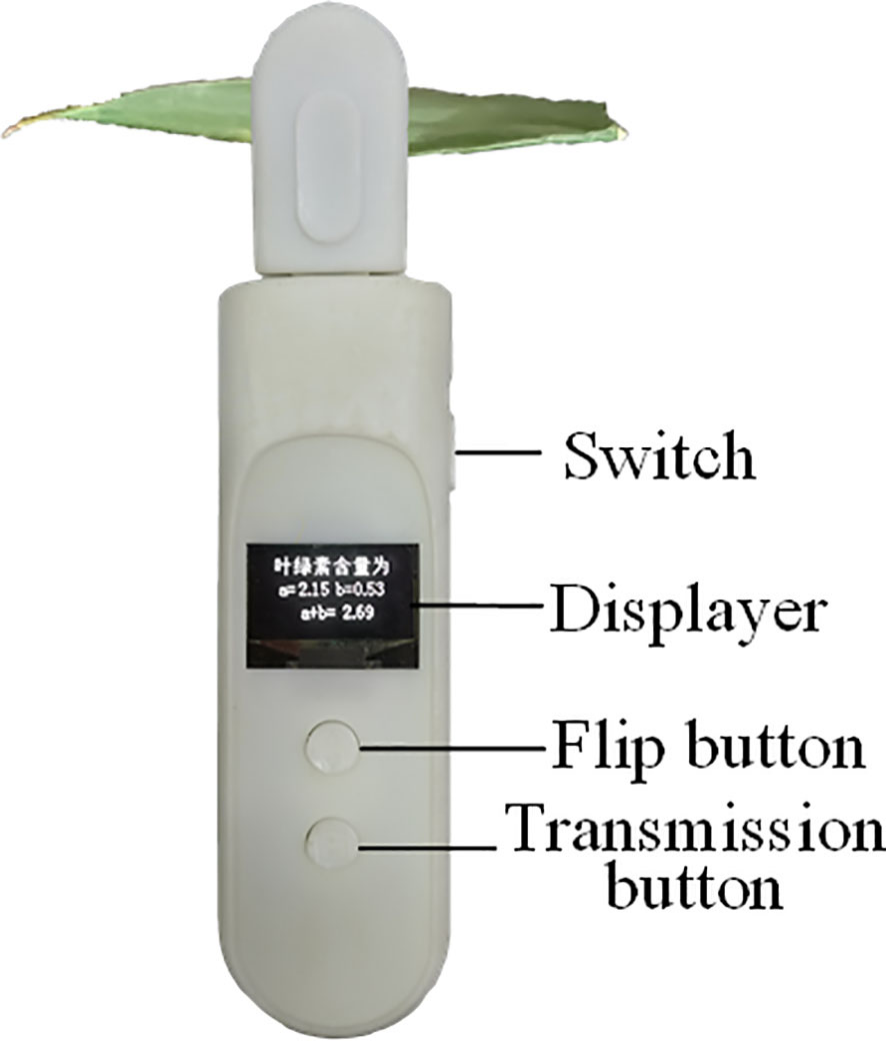
The prototype of the handheld leaf chlorophyll content detector.

**Figure 5:**
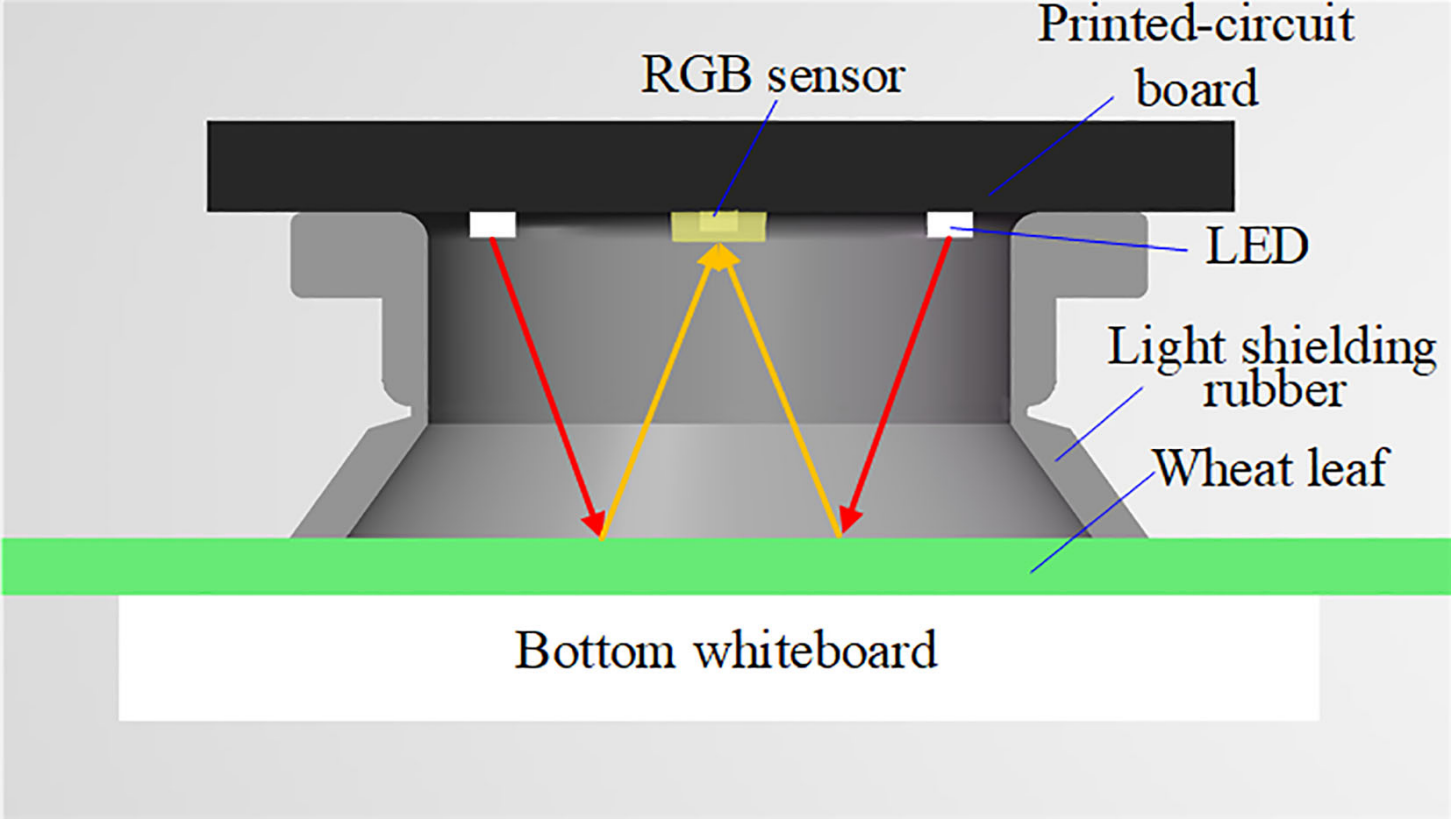
The positions of leaf, LEDs, and RGB sensor.

The main control circuit board is 86 mm×33 mm×1.6 mm in size. The detector shell was designed using SolidWorks software (SolidWorks, Wilmington, USA). The shell can be divided into the instrument body and the detection head. The handheld chlorophyll content detector's prototype is shown in **Figure 4**. Its size is 162 mm×38 mm×29 mm, and its mass is 88 g. A light shielding rubber is added to the detection parts to block external light. When pressing the detection button, the rubber seal contacted the leaf surface closely without damage to the leaves. The positions of the leaf, the LEDs, and the RGB sensors during detection are shown in **Figure 5**.

### 2.2 Software design

**Figure 6:**
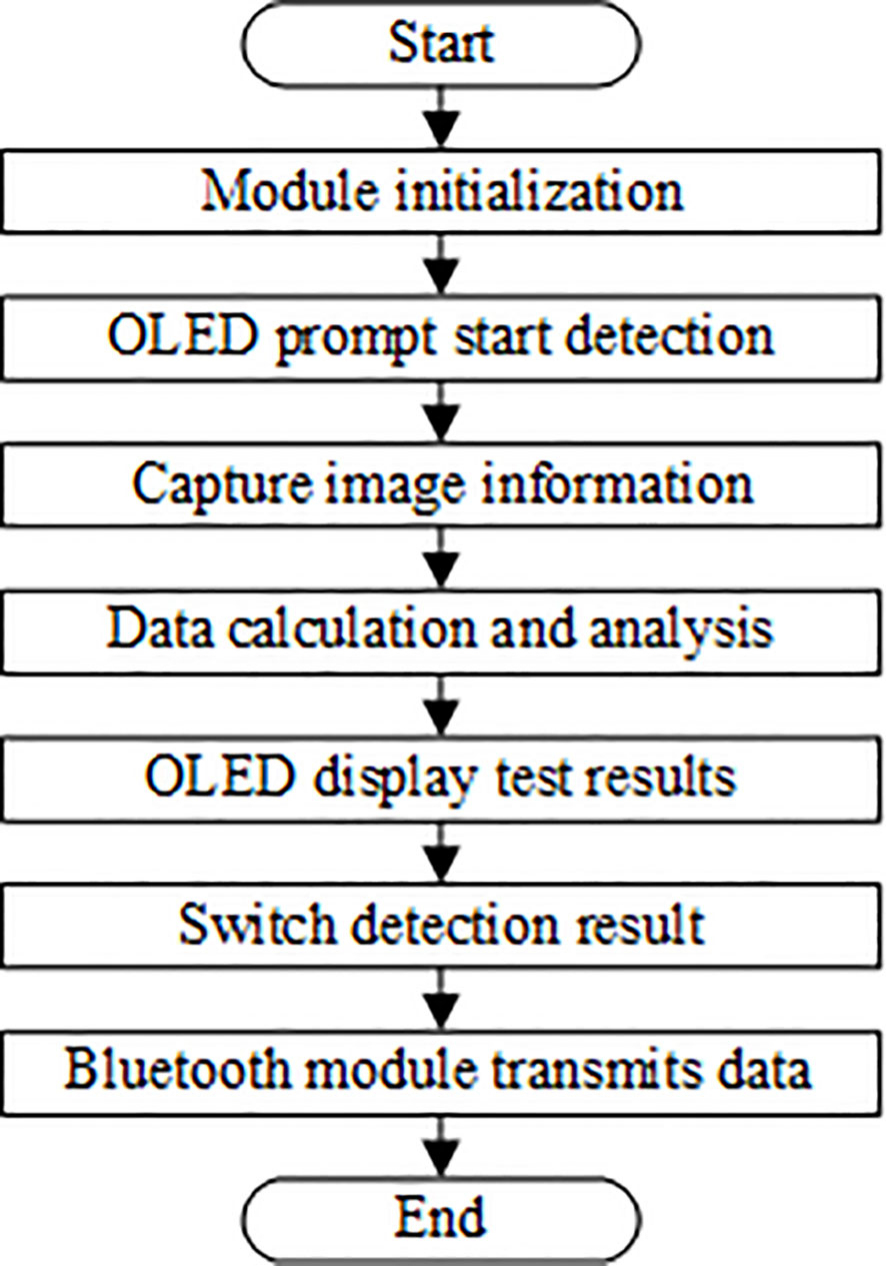
The flow chart of software's running process.

The detector's software, developed in Keil μVision5, consists of a main function and several sub-functions, such as the leaf color collection sub-function, data processing sub-function, key sub-function, and display sub-function. The software's running process is shown in **Figure 6**. After the detector is turned on, each module is initialized, and the OLED displays the welcome interface. After 1 s, the message about measurement is displayed until the detection head is pressed. Then, the RGB sensor collects the color information of the plant leaf and the data is transmitted to the microcontroller for calculation and analysis. If the detection value is abnormal, a detection error is displayed on the OLED screen. Otherwise, the detection results are displayed on the screen. If the transmission button is pressed, the detection results are sent to a mobile phone, and the OLED indicates that the transmission is successful.

## 3 Materials and methods

### 3.1 Materials

The wheat and maize leaf samples used in this study were obtained from different experimental fields at Northwest A&F University, China. The wheat leaves for modeling were collected from 12 wheat cultivars at different experimental plots from May 6 to 10, 2023. There were 71 leaf samples, including 5–6 samples of each cultivar. The maize leaves used for modeling were collected from July 27 to August 1, 2023, and the samples were obtained from four cultivars in different experimental fields, with 15–16 samples of each variety and a total of 61 samples. The samples used in detector performance evaluation were evenly collected from the same experimental fields. The wheat leaves were collected from May 15 to 18, 2023, and the maize leaves were collected from August 5 to 11, 2023. The total samples of wheat and maize were 34 and 31, respectively.

### 3.2 Methods

#### 3.2.1 Collection of color features of crop leaves

After collecting the SPAD values of each plant leaf by using a SPAD-502 chlorophyll detector (Konica Minolta, Japan), the leaf was placed in the detection area of the detector; the detection head was pressed to turn on the LEDs, and then the light intensities of *R*′, *G*′, *B*′ and *C*, output by the RGB sensor, were collected. Based on the obtained *R*′, *G*′, *B*′, and *C*, the color indices of red (*R*), green (*G*), and blue (*B*) were calculated according to Equations 1-3. Then the color indices of *H* (hue), *S* (saturation), and *V* (value) were calculated (Gonzalez and Woods, 2018). To investigate whether other color indices were helpful to improve prediction performance, *R*/*B*, *R*/*G*, *G*/*R*, *G*/*B*, *B*/*R*, *B*/*G*, R*G, R*B, G*B, R*G*B were calculated based on the obtained *R*, *G* and *B*. A triplicate was done for each sample, and the mean of the triplicate was used as the result.


(1) R=RC∗255

(2) G=GC∗255

(3) B=BC∗255

#### 3.2.2 Measurement of the chlorophyll content

After measuring the color features with the developed detector, the chlorophyll extraction solution was prepared using 0.02-0.04 g leaf around the detection points according to Chinese agricultural industry standards of NY/T 3082-2017. The main steps are listed here.

1. An electronic balance with a precision of 0.0001 g (Shanghai JingkeTianmei Scientific Instrument Co., LTD, Shanghai, China) was used to measure the mass of the leaf.
2. The leaf was cut into filaments about 1 mm in width and was placed in a 5 mL volumetric flask. 0.1 mL of pure acetone and 2–3 mL of 80% acetone were added to the volumetric flask.
3. The soaked flask was then kept in a dark room at room temperature and shaken 3–4 times one day. When the leaf turned white two days later, 80% acetone was added to the flask to 5 mL
4. A cuvette (10 mm in distance) was filled with the prepared chlorophyll extraction solution. Then, the absorbance of 80% acetone was used as a contrast to measure the absorbances of chlorophyll extraction solution at 645 and 663 nm using an ultraviolet/visible spectrophotometer (Lambda 365, PerkinElmer Management, Co., Ltd., MA, USA).

Using Arnon's updated equations (Porra et al., 1989), as stated in Equation 4, to calculate the CL-a, CL-b, and TCL contents in solution.


(4) A=KCL

where *K* is the scale factor; *C* is the chlorophyll concentration of the solution, mol/L; *L* is the liquid layer thickness, cm. Detailed information about the calculation of CL-a, CL-b, and TCL contents using the spectrophotometry method could be found elsewhere (Chinese Agricultural Industry Standards, 2017, China.).

#### 3.2.3 Modeling and model evaluation methods

Our previous studies showed that the non-linear models, such as exponential the model, polynomial model and logarithmic models had poor prediction performance than linear models for the CL-a, CL-b, and TCL contents with color features, and the machine learning models, such as support vector regression, random forest, etc, are difficult to be run on the cheap micro-controller of STM32F103C8T6. Therefore, two widely applied linear models, i.e., partial least square regression (PLSR) and multivariate linear regression (MLR) were utilized to regress the relationship between the investigated CL-a, CL-b, and TCL contents with color features in this study. The determination performance of PLSR and MLR were reported in this study, and the performances of regressed PLSR and MLR models were assessed by the coefficients of determination (R^2^) and root-mean-squares error (RMSE).

## 4 Results and discussion

### 4.1 Statistics of chlorophyll contents of wheat and maize leaf samples

**Table 1** presents the statistics of the obtained SPAD values, and the CL-a, CL-b, and TCL contents of 71 wheat and 61 maize leaf samples in modelling and 34 wheat and 31 maize leaf samples in the detector's performance validation.

**Table 1:** Statistics of the chlorophyll contents of used wheat and maize leaf samples.

Plant	Chlorophyll, mg/g	Modeling samples				Validation samples for detector's performance			
		Min.	Max.	Mean ± SD	CV, %	Min.	Max.	Mean ± SD	CV, %
Wheat	SPAD	33.0	65.5	49.3 ± 7.185	14.57	32.9	61.3	47.3 ± 7.570	16.00
	Chlorophyll-a,	1.054	3.442	2.140 ± 0.614	28.68	0.840	3.766	1.977 ± 0.710	35.92
	Chlorophyll-b, mg/g	0.272	1.016	0.571 ± 0.183	32.12	0.212	1.093	0.523 ± 0.207	39.49
	Total chlorophyll, mg/g	1.340	4.415	2.711 ± 0.795	29.32	1.052	4.859	2.499 ± 0.915	36.62
Maize	SPAD	22.8	54.2	37.1 ± 6.605	17.82	23.4	58.8	39.8 ± 7.515	18.88
	Chlorophyll-a,	1.125	2.640	1.828 ± 0.406	22.23	1.088	2.695	1.907 ± 0.439	23.03
	Chlorophyll-b, mg/g	0.217	0.524	0.370 ± 0.084	22.66	0.235	0.526	0.385 ± 0.085	22.07
	Total chlorophyll, mg/g	1.342	3.161	2.198 ± 0.488	22.20	1.329	3.220	2.293 ± 0.523	22.80

Min. is the minimum, Max. is the maximum, SD is the standard deviation, and CV is the coefficient of variation.

It shows that the ranges of TCL contents of wheat and maize leaves used in modeling were 1.340-4.415 mg/g and 1.342-3.161 mg/g, respectively, and were 1.052-4.859 mg/g and 1.329-3.220 mg/g in validation. The coefficient of variation (CV) of the investigated chlorophyll contents of the used leaves in modeling changed from 22.20% to 32.12% and from 22.07 to 39.49% in validation. Generally, the wheat leaves had a wider change range in chlorophyll content than maize leaves. The large CV indicates that the samples used had good representation.

### 4.2 Establishment of models for predicting leaf chlorophyll contents

The 20 color features obtained in Section 3.2.1 were applied as inputs to regress the relationship with CL-a, CL-b, and TCL contents using the PLSR and MLR. The modeling results are listed in **Table 2**. It shows that the MLR model had higher *R* ^2^ and smaller RMSE than PLSR for each chlorophyll content. Therefore, MLR was used to predict chlorophyll content further.

**Table 2:** The prediction performance of established PLSR and MLR models for chlorophyll contents based on 20 color features.

Modeling method	Chlorophyll	*R* ^2^	RMSE, mg/g
PLSR	Chlorophyll-a	0.813	0.274
	Chlorophyll-b	0.789	0.085
	Total chlorophyll	0.817	0.351
MLR	Chlorophyll-a	0.817	0.235
	Chlorophyll-b	0.813	0.076
	Total chlorophyll	0.821	0.304

The significance analysis of the built MLR models for predicting CL-a, CL-b, and TCL contents shows that *B* and *B*′ had very significant relationships (*p*< 0.01) with each chlorophyll content, *H* was correlated with CL-a (*p<* 0.05), *S* and *V* with CL-b (*p*< 0.05), and *H* and *S* with TCL (*p*< 0.05) significantly, shown in **Table 3**. The relationships of other color features with these chlorophyll contents weren't significant (*p* > 0.05). To eliminate redundant parameters, reduce the complexity of the models, and avoid overfitting, the MLR models for predicting CL-a ( $W_{a}$ ), CL-b ( $W_{b}$ ), and TCL content ( $W_{t}$ ) were built using the 5 color features, that is, *B*, *B*′, *H*, *S*, and *V*. The models are shown in Equations 5-7. The *R* ^2^ of the built models for CL-a, CL-b, and TCL contents were 0.796, 0.795, and 0.801, respectively, and the RMSEs were 0.248 mg/g, 0.080 mg/g, and 0.320 mg/g. Although the modeling performances are a little poorer than those of the built models using the 20 color features (**Table 2**), the models are much simpler.

**Table 3:** The significance of 5 color features to the built MLR models for predicting chlorophyll contents of wheat and maize leaves.

Chlorophyll	*B*	*B*′	*H*	*S*	*V*
Chlorophyll-a	<0.001	0.004	0.012	0.060	0.087
Chlorophyll-b	<0.001	0.008	0.105	0.019	0.021
Total chlorophyll	<0.001	0.003	0.018	0.040	0.057


(5) $$\begin{array}{c} W_{a}=-30.623+1.143\times B-0.225\times B^{\prime }+0.008\times \\ H+85.096\times S-170.899\times V,\;\;R^{2}=0.796 \end{array}$$

(6) $$\begin{array}{c} W_{b}=-13.164+0.467\times B-0.052\times B^{\prime }+0.005\times \\ H+35.536\times S-72.08\times V,\;\;\;R^{2}=0.795 \end{array}$$

(7) $$\begin{array}{c} W_{t}=-43.787+1.609\times B-0.276\times B^{\prime }+0.013\times \\ H+120.632\times S-242.979\times V,\;\;\;R^{2}=0.801 \end{array}$$

### 4.3 Evaluation of the developed detector's repeatability and stability

The repeatability of the developed detector in obtaining color features was evaluated by randomly selecting three wheat leaves and three maize leaves with different chlorophyll contents. The *R′*, *G′*, *B′*, and *C* of each leaf were measured 10 times on the same point. The mean, standard deviation, and CV of each color index for each sample are listed in **Table 4**. It shows that the CV ranges from 0.000% to 2.083% with a mean of 0.516%, indicating that the detector has good repeatability in obtaining color features.

**Table 4:** Statistics of color features at 10 times measurements for randomly selected 3 wheat and 3 maize leaves.

Sample number	Plant	*R*′		*G*′		*B*′		*C*	
		Mean ± SD	CV, %	Mean ± SD	CV, %	Mean ± SD	CV, %	Mean ± SD	CV, %
1	Wheat	27.9 ± 0.0	0.000	27.0 ± 0.1	0.341	18.8 ± 0.1	0.382	80.9 ± 0.5	0.666
2		29.0 ± 0.0	0.000	29.0 ± 0.0	0.000	19.2 ± 0.4	2.083	85.0 ± 0.0	0.000
3		25.8+0.0	0.000	23.8 ± 0.0	0.000	18.0 ± 0.0	0.000	74.0 ± 0.0	0.000
4	Maize	29.9 ± 0.3	1.003	26.5 ± 0.5	1.889	19.0 ± 0.0	0.000	82.1 ± 0.5	0.656
5		30.9 ± 0.3	0.971	27.9 ± 0.3	1.075	20.9 ± 0.3	1.435	86.3 ± 0.6	0.742
6		30.0+0.0	0.000	26.1 ± 0.3	1.149	20.0 ± 0.0	0.000	78.0 ± 0.0	0.000

SD is the standard deviation, CV is the coefficient of variation, and the unit of *R*′, *G*′, *B*′ and *C* are counts/μW/cm2.

As chlorophyll content may exhibit slight diurnal variation even within the same leaf and pot, the stability of the developed detector in capturing color features was evaluated using several color cards of different hues. Measurements were conducted in a maize field on a sunny day at 8:00, 12:00, 16:00, and 20:00. The data are listed in **Table 5**. The CV of the mean *R′*, *G′*, *B′*, and *C* values at different times were 0.911%, 1.103%, 0.941%, and 0.771%, respectively, indicating that the detector has good stability.

**Table 5:** Statistics of color features on several color cards at different time in a sunny day outside.

Time	Temperature (°C)	Humidity (%)	*R*′		*G*′		*B*′		*C*	
			Mean ± SD	CV, %	Mean ± SD	CV, %	Mean ± SD	CV, %	Mean ± SD	CV, %
8:00	22.3	55.1	69.2 ± 0.4	0.578	39.0 ± 0.0	0.000	36.2 ± 0.4	1.104	155.6 ± 0.5	0.321
12:00	24.9	53.8	71.0 ± 0.0	0.000	40.0 ± 0.0	0.000	37.0 ± 0.0	0.000	159.0 ± 0.0	0.000
16:00	29.8	44.7	70.0 ± 0.0	0.000	39.0 ± 0.0	0.000	37.0 ± 0.0	0.000	157.0 ± 0.0	0.000
20:00	22.7	52.4	70.0 ± 0.0	0.000	39.0 ± 0.0	0.000	37.0 ± 0.0	0.000	157.0 ± 0.0	0.000

SD is the standard deviation, CV is the coefficient of variation.

### 4.4 Evaluation of the developed detector's precision

After the software with the MLR model for predicting chlorophyll contents was downloaded to the explored detector, the precision of the explored detector was evaluated using the 34 wheat and 31 maize leaves in the validation set (**Table 1**). It is noted that the used samples had a wide range of chlorophyll contents. After the chlorophyll content of each leaf was measured using the detector, the actual chlorophyll contents of each leaf were measured according to the method given in Section 3.2.2.

**Figure 7:**
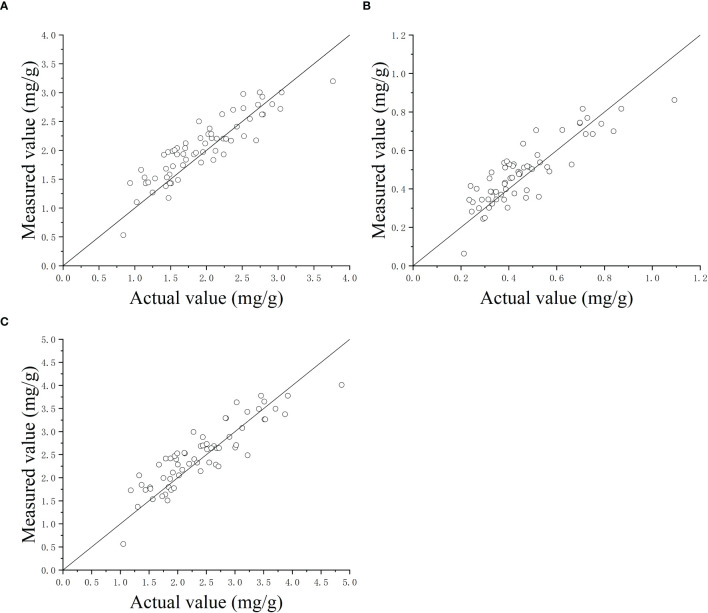
The detected contents of chlorophyll-a **(A)**, chlorophyll-b **(B)**, and total chlorophyll **(C)** using the detector versus the actual values measured using spectrophotometry method.

**Figure 7** shows the detected CL-a, CL-b, and TCL contents using the explored detector against the measured values using spectrophotometry. The symmetrical distribution of points around the 45° line suggests that the detector demonstrates high precision. The RMSEs of the explored detector were 0.269 mg/g for CL-a, 0.089 mg/g for CL-b, and 0.350 mg/g for TCL contents of wheat and maize leaves, respectively, and had the mean absolute errors of 0.104 mg/g, 0.031 mg/g, and 0.087 mg/g.

In order to evaluate the predictive performance across crop species, separate analyses were conducted for wheat and maize leaves. The RMSE values for CL-a, CL-b, and TCL in wheat were 0.264 mg/g, 0.090 mg/g, and 0.346 mg/g, respectively, while those for maize were 0.271 mg/g, 0.083 mg/g, and 0.347 mg/g. These results demonstrate that the predictive performance was generally consistent between the two crops. This similarity may lie in their shared phylogenetic traits as *Poaceae* plants, which exhibit analogous chlorophyll spectral absorption characteristics. Additionally, their parallel leaf venation and similar epidermal structures likely lead to nearly identical light scattering and absorption pathways within leaves.

### 4.5 Discussion

Li et al. (2021) used an auxiliary device to assist smartphones in shooting RGB images of sugarcane leaves to detect their chlorophyll content. They established a support vector machine regression model to predict the chlorophyll content with an average RMSE of 0.3288 mg/g, a little lower than the obtained 0.350 mg/g for chlorophyll content here. However, the image color is affected by many factors, such as light, camera used in different smartphones, shooting angles, etc., restricting the application of this method. Moreover, the contents of CL-a and CL-b cannot be given.

The TCL content detector developed by Yang et al. (2019) had an absolute measurement error of -0.32-0.20 mg/g and a mean absolute error of 0.14 mg/g for spinach, big green vegetables, and lettuce leaves. However, only TCL can be measured. In contrast with their works, the obtained RMSEs here are higher. The reason might lie in several aspects. One reason is that the wheat leaf is very narrow, which causes the obtained color features to contain the information of leaf veins and make the color features, especially *B*′ (which has a significant correlation with each chlorophyll content), to have a wide variation, lowering the precision. Another is that the wheat and maize leaves had much higher chlorophyll content and wider change ranges than the leafy vegetables they used. The third is that the samples used herein validation had a wider change range in chlorophyll content than those used in modeling, also decreasing the precision. In addition, although the cheap RGB sensor (about 2 US dollars) decreases the detector's cost, it also reduces the precision. Using high-performance RGB sensors would help improve the precision determination of CL-a, CL-b, and TCL contents.

To evaluate whether the color indices are more efficient in determining chlorophyll content than SPAD values, the linear relationship between SPAD values and the chlorophyll content was analyzed. The RMSE of predicting TCL content in wheat and maize leaves using SPAD values was 0.428 mg/g. The results indicate that the device developed in this study exhibits better performance in predicting chlorophyll content in wheat and maize leaves than the SPAD-502 chlorophyll detector. The developed detector in this study has a mass of only 88 g. It can give the test results within 2 s without destruction to crop leaves, meeting the requirements of real-time and non-destructive detection of chlorophyll contents of crop leaves. However, since the current model is specifically established for wheat and maize, further research is required to evaluate its applicability to other crop species.

## 5 Conclusions

Based on the significant correlation between chlorophyll contents and leaf color features, a detector was designed to non-destructively detect the CL-a, CL-b, and TCL contents of wheat and maize leaves. The detector mainly comprises a microcontroller, light source, RGB sensor, power supply, Bluetooth transmission, and other modules. The relationship between chlorophyll contents and color features of wheat and maize leaves can be expressed by linear equations in five variables, in which *B* and *B*′ had very significant relationships and *H*, *S*, and *V* had significant relationships with each chlorophyll content. The RMSEs of the detector were 0.269 mg/g for CL-a, 0.089 mg/g for CL-b, and 0.350 mg/g for TCL contents of wheat and maize leaves. The detection results could be given within 2 s without destruction to leaves. This study provides a protocol for non-destructive, real-time, low-cost, and rapid detection of chlorophyll contents in wheat and maize leaves. The feasibility of this method in detecting chlorophyll contents of other plants and the precision improvement method will be studied in the future.

## Data Availability

The original contributions presented in the study are included in the article/supplementary material. Further inquiries can be directed to the corresponding authors.
